# PROVIDERS, FUNCTIONS, AND ADEQUACY OF CARE AMONG CENTENARIANS: INSIGHTS FROM SWISS100

**DOI:** 10.1093/geroni/igae098.0218

**Published:** 2024-12-31

**Authors:** Barbara Masotti, Daniele Zaccaria, Francois Herrmann, Daniela Jopp, Armin von Gunten, Stefano Cavalli

**Affiliations:** University of Applied Sciences and Arts of Southern Switzerland, Manno, Ticino, Switzerland; University of Applied Sciences and Arts of Southern Switzerland, Manno, Ticino, Switzerland; Geneva University Hospitals, Thônex, Geneve, Switzerland; University of Lausanne, Lausanne, Vaud, Switzerland; Lausanne University Hospital, Prilly, Vaud, Switzerland; University of Applied Sciences and Arts of Southern Switzerland, Manno, Ticino, Switzerland

## Abstract

At age 100 and beyond, individuals deal with increasing support needs, especially when their social network is at risk due to the death of spouses, siblings, and friends, while children may face own age-related health issues. However, little is known about the specific aids received by centenarians. Drawing from the care convoy framework, this study explored the diversity of care among Swiss centenarians. We investigated the composition and the functions of their care convoys and analysed the impact of individual and contextual characteristics. Additionally, we examined participants’ perception of the adequacy of the aids received. The analyses are based on centenarian and/or proxy reports from the study SWISS100 (N = 275; Mage = 101.789) using descriptive statistics and logistic regression models. Subjective adequacy is examined through qualitative content analysis. Findings indicate that centenarians are part of intricate convoys of care. The diversity of types of care and providers was high among community-dwelling, where informal and formal caregivers both provide help with a wide range of tasks (e.g., personal care, monitoring). In institutional settings there was a more defined division of roles, with family and friends providing administrative and socioemotional support. The impact of individual and contextual factors varied depending on the type of care and/or the care provider. Participant satisfaction did not always align with the quantity of care received. Considering, in conclusion, the diversity of care observed, understanding centenarians’ needs and resources is essential for developing tailored policies and services for them and their families.

